# Referential communication in people with recent-onset schizophrenia-spectrum disorders

**DOI:** 10.3389/fpsyt.2022.971256

**Published:** 2022-09-09

**Authors:** Amélie M. Achim, André Achim, Marion Fossard

**Affiliations:** ^1^Département de Psychiatrie et Neurosciences, Université Laval, Québec, QC, Canada; ^2^Centre de Recherche CERVO and Centre de Recherche VITAM, Québec, QC, Canada; ^3^Département de Psychologie, Université du Québec à Montréal, Montréal, QC, Canada; ^4^Institut des Sciences Logopédiques, Université de Neuchâtel, Neuchâtel, Switzerland

**Keywords:** referential communication, verbal production, collaboration, communication, social interactions, psychosis

## Abstract

People with schizophrenia present with language production impairments, yet very few studies examine language production in the context of collaborative, verbal interaction tasks performed with a real interaction partner. The current study relied on a referential communication paradigm in which participants with schizophrenia (SZ) and healthy controls (HC) presented a series of movie characters to their interaction partner, whose role was to identify and place the characters in the same order. The HC spontaneously provided more information when presenting characters that their interaction partner was unlikely to know than when presenting very well-known characters, and the magnitude of this adjustment was positively correlated with their performance on a theory of mind task. In contrast, people with SZ showed a significantly reduced (absent) adjustment to the likely-known vs. likely-unknown nature of the characters, and no correlation emerged with ToM. Further examination of the verbal productions revealed that HC often combined movie-related information (ex: character's name or movie title) and descriptive information whereas people with SZ more often used description only to present the characters. Overall, this study adds to our knowledge about referential choices in SZ in the context of collaborative verbal interactions with a real interaction partner.

## Introduction

People with schizophrenia present with a range of cognitive and behavioral impairments, including difficulties with language production (1–3). The language production deficits in schizophrenia are observed particularly at the discourse level, whereas the ability to produce words or to produce sentences with appropriate syntax is generally spared (4).

At the discourse level, it is clear that people with schizophrenia can present with symptoms of thought disorder (5–7), which are in actuality deficits in language production believed to reflect disorganized thoughts. Thought disorder symptoms (e.g., incoherence, tangentiality, derailment, etc.) are typically rated by a clinician or research assistant based on the observed behavior of the patient during clinical or research interviews. Hundreds of studies have reported thought disorder symptoms in people with schizophrenia, these symptoms being generally more important in the acute phase of the illness and typically less pronounced in stabilized outpatients (8, 9).

Another line of research revealed an increased rate of referential failures in the discourse of people with schizophrenia (2, 10–12). More specifically, the referential failure approach consists of the identification of instances of unclarities (e.g., the use of a reference in speech for which the referent, i.e., the person or object being referred to, is unclear, ambiguous, or not previously introduced, which impairs the understandability of the message) in free speech samples for which rates of unclarities are calculated per 100 words. This approach was used in dozens of studies that together confirmed the higher rate of reference failures in people with schizophrenia compared to healthy controls (2, 10–12).

Beyond these pioneering approaches, the linguistic approach to studying the language production deficits in schizophrenia has gained increased attention as it opens novel avenues to understanding the difficulties that people with schizophrenia face during verbal interactions (13–17). Studies based on the linguistic approach can be divided into two categories: (1) studies using automated analysis software that can be applied to a large corpus of speech samples (free speech or interview transcripts) (15, 18); (2) studies relying on experimental linguistic procedures, with controlled yet naturalistic experimental tasks designed to examine specific aspects of language production [e.g., (13, 14)]. One advantage of using experimental linguistic procedures to generate the speech samples is that we can further examine the contexts (tasks or task conditions) in which communication impairments are more likely to emerge.

Despite its potential, surprisingly few studies have relied on the experimental linguistic approach to examine speech production in people with schizophrenia. Two studies asked participants with schizophrenia and healthy controls to perform a storytelling task and then extracted different linguistic features from the speech samples (19, 20). These studies notably revealed local and global coherence errors (failure to link utterances conceptually) in people with schizophrenia. However, the tasks used for these studies focused on the production of monologs, rather than interactions or dialogues, and did not include a manipulation of task conditions.

One experimental linguistic paradigm that is particularly well-suited for the examination of language production during real social interactions is the referential communication paradigm, which has already been used in a large number of experimental linguistics studies in healthy participants (21–25). The referential communication paradigm reproduces a communication situation involving a social interaction based on the collaboration between two partners: (1) the participant, who must present a series of images to his partner, and (2) the matcher, who has to identify the images and place them in the correct order based on the instructions provided by the participant. This type of task allows for real verbal interactions between the two partners since the matcher can provide feedback, ask for clarifications, and exchange with the participant during the task. In addition, the images being presented by the participant can be manipulated to identify the conditions in which different linguistic features are more likely to emerge, or the conditions under which the verbal productions of patients with different diagnoses are particularly affected.

In one of the few studies to use the referential communication paradigm to examine speech production in people with schizophrenia, participants were asked to describe two sets of tangram figures (abstract shapes) to the experimenter so that the experimenter could place the five figures of each set in the correct order (13). The procedure was repeated 5 times with each of the two sets of tangram figures, introducing a manipulation of the turn (first turn vs. subsequent turns, with the images in a different order for each turn). The authors focused their analyses on the use of either indefinite (e.g., “a mountain”) or definite (e.g., “the mountain”) reference markers, and patients and controls did not significantly differ in their use of these markers when considering specifically the first turn of the task. The groups however differed on subsequent turns, such that the healthy participants started using a lesser proportion of indefinite references [typically expected when items are first introduced in the discourse (23)] and a greater proportion of definite references [typically expected when items are reintroduced again at a later point in the exchange (23)], while people with schizophrenia continued using a high proportion of indefinite references throughout the task (and hence less definite references). This suggests that in their verbal productions, people with schizophrenia failed to mark the status of the referents as either new (when first introducing a referent) or given (i.e., previously mentioned, when later reintroducing the same referent), as healthy participants do through the use of distinct reference markers (21, 23, 26, 27).

Interestingly, this pattern in schizophrenia was even more pronounced when considering the patients who were also impaired on a theory of mind (ToM) task, as compared to those without a ToM deficit (13). Theory of mind refers to the ability to infer or represent the mental states of other people, and some authors have proposed that it could play a role in helping us adjust our speech in order to facilitate understanding for our interaction partners (13, 22, 28).

For instance, a recent referential communication study (21, 22) revealed that healthy participants do adjust their reference choices depending on the knowledge that their interaction partner can be expected to have about the material being discussed (e.g., pictures of movie characters that were either very well-known or less known), even on the first presentation of the material. In addition, healthy participants with better ToM showed a greater adjustment in the way they presented the movie characters in that study, using more pieces of information to present the characters that the partner was less likely to know (e.g., “it is Leonidas from the movie 300, he has a black beard and a red cape”) and less elements of information for characters that the partner was very likely to know (e.g., “it's Harry Potter”) (22).

The current study aimed to use the same task based on movie characters to determine if participants with schizophrenia also adjust the way they present movie characters that are more or less likely to be known by their interaction partner. Given that these adjustments seem linked to ToM abilities according to Achim et al. (22) and given that people with schizophrenia often present with ToM deficits (29, 30), we expected that our schizophrenia group would show a reduced adjustment in the way they present characters that their interaction partner is very likely vs. less likely to know.

## Methods

### Participants

We aimed to recruit twenty five (25) outpatients with recent-onset schizophrenia spectrum disorder from the Clinique Notre-Dame des Victoires, a specialized clinic for first-episode psychosis in Quebec City, Canada. The targeted DSM-IV diagnoses included schizophrenia, schizoaffective disorder, delusional disorder, and psychosis not otherwise specified.

We also aimed to include twenty five (25) healthy controls matched to the outpatients in terms of age, gender, and parental socioeconomic status as assessed with the Hollingshead two-factor index of social position (31). Healthy controls were recruited through ads and pamphlets circulated at Laval University, public places, social media and word of mouth.

The inclusion/exclusion criteria for both groups were to have French as their first or main language (having studied in French for elementary school and high school was accepted) and to have no history of a neurological disorder or head trauma. Additional criteria for the healthy controls were to have never received a diagnosis for a mental disorder and no current use of psychoactive medication.

This project was approved by the Research Ethics Board of the CIUSSS-CN—neuroscience and mental health division, and all participants signed an informed consent.

### Procedure

Because our experimental task is based on movies (see below), participants first completed a movie knowledge questionnaire, then completed a social cognition assessment (as well as additional tests and questionnaires not reported here) and finally performed our experimental task.

### Experimental task

**Figure 1:**
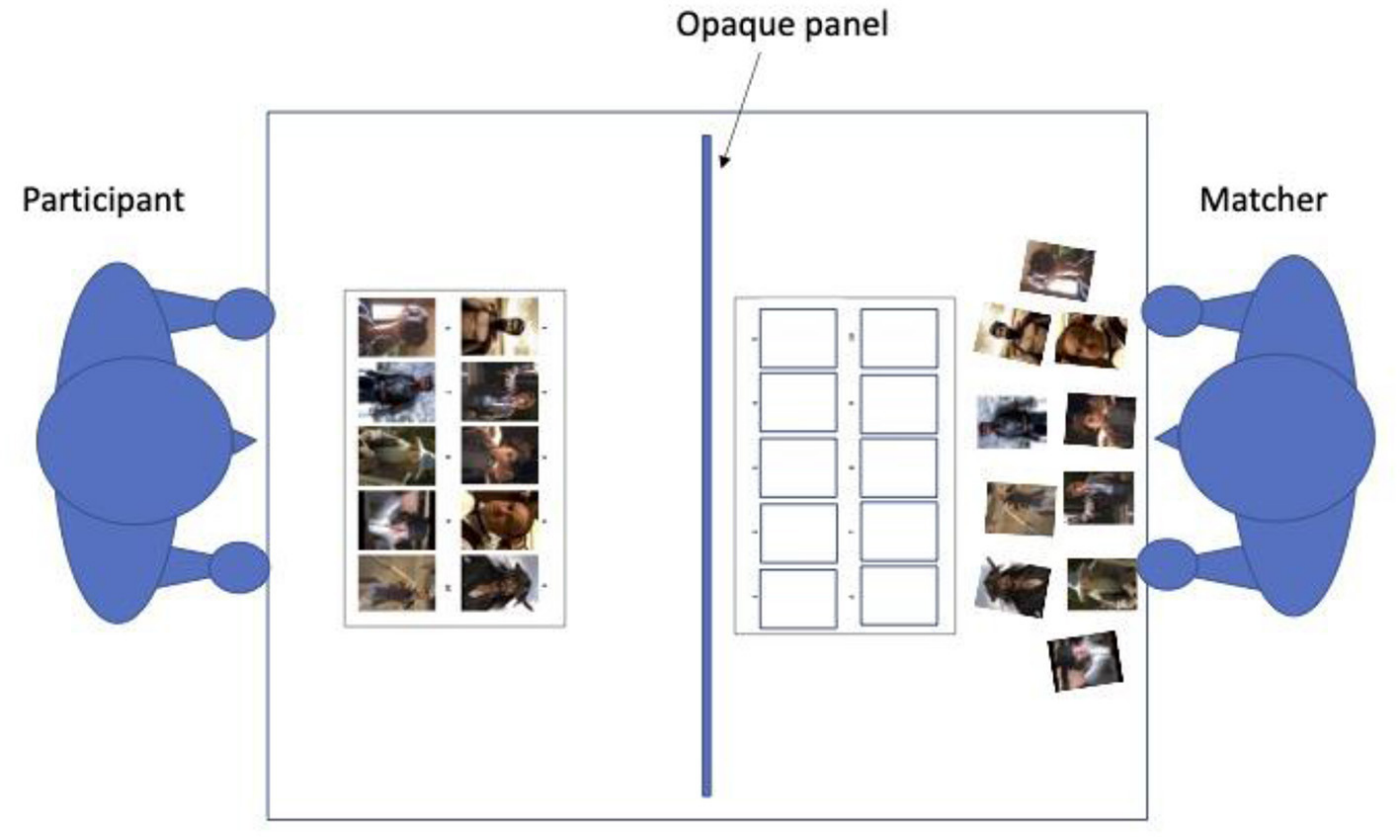
Illustration of the experimental task.

The current project is based on the referential communication paradigm [e.g. (13, 23, 25, 32)] and the experimental task is the same as that used in Achim et al. (22). Before the beginning of the task, an opaque panel is placed between the participant and his addressee, i.e., the matcher. As illustrated in Figure 1, the participant receives a sheet with a series of 10 images representing movie characters, presented in a predetermined order on the sheet. The matcher receives the same 10 images, but on separate cards and in a random order. In addition, the matcher also receives a response sheet with 10 empty spaces on which to place the images during the task.

The role of the participant is to present each character one at a time so that the matcher can place the images in the same order. Participants are requested not to name the actors, and to focus rather on the characters. The role of the matcher is to place the images in order on their response sheet and to ask the participant for clarifications if needed.

A manipulation was introduced such that half of the images represented characters that most women in their twenties in Quebec should know according to a series of surveys [the Likely-Known condition; see (22) for more details], whereas the other half of the images represented characters that a much smaller proportion of these women would know (the Likely-Unknown condition). Accordingly, the matcher was always a woman in her twenties, and this role was held by a trained research assistant to standardize the feedback given for the different items in the card set. The matcher knew all the characters in the Likely-Known condition and could hence identify these characters based on their name, the movie title, information about the role of the character in the movie or descriptive information. For the Likely-Unknown condition, the matcher was trained to act as if she did not know any of the characters in that condition. More specifically, when participants were presenting the Likely-Unknown characters, she was trained to rely only on descriptive information (e.g., “has a black beard”) to identify and place the images, and to disregard any movie-related information such as the name of the character (e.g., “Leonidas”) or the title of movie (e.g., “from the movie 300”). The material and procedure were created so that participants had to take into consideration the knowledge that they can attribute to the addressee for each of the different movie characters (i.e., which movie characters are likely known by their addressee) on an image-by-image basis in order to produce appropriate referential information.

The participants were aware that the matcher was a research assistant, and a concealment strategy was developed to make the participants believe that the assistant/matcher did the task with different images for each participant (when in fact the same 10 images were always used). More specifically, the images were presented in a sealed envelope allegedly prepared by another research assistant and participants were told that the matcher had previously done the task but each time with a different set of images, so they had not been previously exposed to the images that were in the envelop. A pilot study previously confirmed the success of this strategy, with none of the 10 pilot subjects reporting suspecting that the assistant/matcher was familiar with the material used for the task.

### Movie knowledge questionnaire

The movie-knowledge questionnaire included images of the 10 movie characters used in the experimental task, and for each character, participants had to report whether they had seen the movie or not. Participants who reported seeing the movie were also asked how long ago they had seen it and to estimate the percentage of women in their twenties in Quebec they thought had also seen the movie.

### Theory of mind assessment

Theory of Mind was assessed with the Combined Stories Task (COST), a validated ToM task with good psychometric properties (29, 33). For this task, participants read a series of 29 short stories, and for each story they are asked to answer 2 or 3 open questions. The questions target the mental states of the story characters (ToM questions, 26 questions), physical causalities (non-social reasoning questions, 6 questions) or details of the stories (control questions, 29 questions). The answers to the ToM questions are scores 0, 1 or 2 points according to a validated scoring grid and are summed up to get the total ToM score (/52).

### Data processing and analyses

For the experimental task, the verbal productions were transcribed and subsequently coded to identify, for each trial (i.e., each character that was presented), all the pieces of information that were spontaneously provided (before any clarification requested from the matcher/experimenter) and their type: (1) *Descriptive information*: any piece of information that could lead to a visual identification of the character, such as their physical attributes (ex: “has a beard”), clothing (ex: “white shirt”), visually explicit roles (ex: “it's an extraterrestrial”), etc.; (2) *Movie related information*: any piece of information related to the character or the movie, including the name of the character, the name of the movie or the role of the character in the movie (only for non-visual roles, for example saying “he's a cop” for a character that is not dressed as a policeman). In a second step, for each character that the participants themselves knew, we calculated the number of pieces of information used to present the character, regardless of the type of information (this is the measure for which we expected a lesser adjustment in people with SZ given its link with ToM in healthy participants) (22). For example, a character presented using their name and two pieces of descriptive information (e.g. “it is Leonidas, he has a red cape and a beard”) is presented with a total of three pieces of information whereas a character presented using only their name (e.g., “it is Leonidas) is presented with a total of one piece of information. In a third step, for each participant we also classified each character that they knew according to whether they were presented using: (1) movie related information only; (2) a combination of movie related information and descriptive information; (3) descriptive information only. As in Achim et al. (22), the characters that the participants did not know were excluded from the analyses to fully focus on the effect of the likely knowledge of the matcher. All condition counts were thus expressed as their proportions of the retained characters.

Each of the four variables created in the second and third step above were submitted to a group (SZ, HC) by condition (Likely-Known, Likely-Unknown) ANOVA. Given that our variables were not all normally distributed (e.g., there were few cases where participants presented Likely-Known characters using only descriptive information, leading to a floor effect), we re-examined the probabilities of our different effects (effects of group, condition and interaction) with a series of *t*-tests (corresponding to each effect of the ANOVA) for which the probability of the observed *t*-values was assessed through simulations. More specifically, we determined the rank of the observed *t*-value among the distribution of *t*-values obtained for 1,000 random reassignments of group membership or of signs to the observed differences (raised to 20,000 reassignments when the observed probability was within the 95% confidence interval of the alpha threshold of *p* < 0.05). Given that these probabilities did not change the pattern of results, we chose to report the standard *p*-values (based on the normal distribution) in the results section, with increased confidence that these probabilities were reliable with the current distributions.

## Results

### Participants

Twenty-five (25) outpatients with recent-onset schizophrenia spectrum disorder were successfully recruited, including patients with schizophrenia (*N* = 17), schizoaffective disorder (*N* = 6) and delusional disorder (*N* = 2). The duration of illness ranged from 5 to 67 months, with an average of 26.2 months (SD = 17.5).

We recruited twenty-five (25) healthy controls participants that did not significantly differ from the SZ group with respect to age, gender and parental socioeconomic status. Healthy participants were a subset of those included in Achim et al. (22), and the data for both groups were acquired within the same time period. Additional sociodemographic and clinical information is presented in Table 1.

**Table 1:** Sociodemographic and clinical data.

	**Schizophrenia**	**Healthy controls**	**Between-group effects**
Gender (male/female)	22/3	20/5	$X_{\left(1\right)}^{2}$ = 0.60, NS
Age (mean, SD)	27.0 (4.6)	25.1 (5.8)	*t*_(48)_ = 1.28, NS
Parental socioeconomic status (Mean, SD)	3.5 (1.0)	3.2 (0.9)	*t*_(47)_ = 1.10, NS
ToM performance on the COST	37.4 (8.0)	43.0 (5.0)	*t*_(48)_ = 2.93, *p* = 0.005
**PANSS 5 factors (mean, SD)**			
PANSS positive	14.6 (4.9)	—————	—————
PANSS negative	16.0 (6.3)	—————	—————
PANSS cognitive/disorganization	9.4 (3.3)	—————	—————
PANSS depression/anxiety	8.3 (2.6)	—————	—————
PANSS excitability/hostility	5.8 (2.3)	—————	—————
PANSS total (mean, SD)	61.6 (16.9)	—————	—————
SOFAS (mean, SD)	56.5 (11.9)	—————	—————
**Antipsychotic medication**			
Aripiprazole	*N* = 9	—————	—————
Risperidone or risperidone consta	*N* = 5	—————	—————
Quetiapine XR	*N* = 5	—————	—————
Clozapine	*N* = 3	—————	—————
Olanzapine	*N* = 1	—————	—————
Paliperidone	*N* = 1	—————	—————
Combination aripiprazole + quetiapine	*N* = 1	—————	—————
**Other medications**			
Antidepressant	*N* = 5	—————	—————
Benzodiazepines	*N* = 8	—————	—————
Lithium	*N* = 1	—————	—————
Valproic acid	*N* = 1	—————	—————
Lamotrigine	*N* = 5	—————	—————

NS, Not statistically significant; SD, Standard deviation; ToM, Theory of mind; COST, Combined Strories Task (29, 33); PANSS, Positive and Negative Symptoms Scale (34); SOFAS, Social and occupational functioning assessment scale (35).

### Results from the movie questionnaire

As shown in Table 2, the groups did not significantly differ in terms of the number of movies that the participants had themselves seen according to the movie knowledge questionnaire.

**Table 2:** Number of movies from the experimental task that the participants had seen.

	**Schizophrenia**	**Healthy controls**	**Between-group effects**
Total number of movies that the participants had seen (/10)	7.04 (1.97)	7.36 (1.87)	*t*_(48)_ = 0.59, *p* = 0.56
Number of likely-known movies that the participants had seen (/5)	4.16 (0.94)	4.56 (0.71)	*t*_(48)_ = 1.70, *p* = 0.10
Number of likely-unknown movies that the participants had seen (/5)	2.88 (1.33)	2.80 (1.63)	*t*_(48)_ = −0.19, *p* = 0.85

### Results for the number of pieces of information

**Figure 2:**
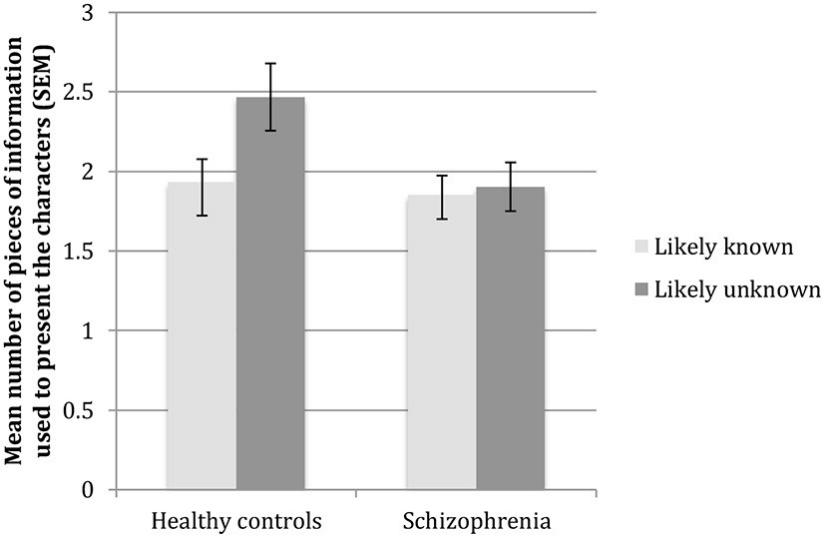
Number of pieces of information provided by the participant to present each character.

The results for the number of pieces of information provided by participants are presented in Figure 2. The analyses revealed a significant effect of conditions [*F*_(1, 44)_ = 6,02, *p* = 0.018] and a significant interaction between group and condition [*F*_(1, 44)_ = 4.11, *p* = 0.049], while the main effect of group did not reach significance [*t*_(44)_ = 2.78, *p* = 0.103]. The interaction reflected that the effect of condition was significant in the healthy control group [*t*_(22)_ = 2.66, *p* = 0.014] but not in the schizophrenia group [*t*_(22)_ = 0.39, *p* = 0.698].

When looking at the association between how much each participant adjusted the number of information that they provided between the two conditions (Likely-Unknown minus Likely-Known) and their ToM performance on the COST, a significant positive association was observed in the healthy control group (*r* = 0.45, *p* = 0.032), such that participants with better ToM showed greater adjustments. This association did not reach significance in the schizophrenia group (*r* = −0.22, *p* = 0.323).

### Results for the type of information

**Figure 3:**
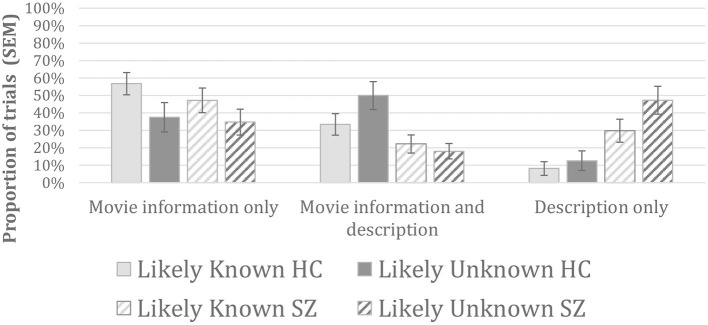
Type of information used by the participants to present each character.

The results regarding the type of information used by the participants to present the characters are presented in Figure 3.

The analyses targeting the trials for which participants used only movie-related information revealed a main effect of condition [*F*_(1, 44)_ = 9.39, *p* = 0.004], with a greater proportion of trials for the Likely-Known than for the Likely-Unknown characters. The main effect of group [*F*_(1, 44)_ = 0.46, *p* = 0.499] and the interaction [*F*_(1, 44)_ = 0.43, *p* = 0.514] were not statistically significant.

The analyses targeting the trials for which participants used both movie information and descriptive information revealed a main effect of group [*F*_(1, 44)_ = 10.86, *p* = 0.002], with a greater proportion of trials for the control group relative to the SZ group. The effect of condition [*F*_(1, 44)_ = 1.234, *p* = 0.273] and the interaction [*F*_(1, 44)_ = 3.43, *p* = 0.071] did not reach significance.

The analyses targeting the trials for which participants used only descriptive information also revealed a main effect of group [*F*_(1, 44)_ = 14.14, *p* < 0.001], this time with a greater proportion of trials of this type for the SZ group relative to the control group, and a main effect of condition [*F*_(1, 44)_ = 5,73, *p* = 0.021], with a greater proportion of trials for the Likely-Unknown condition relative to the Likely-Known condition. The interaction between groups and conditions did not reach significance [*F*_(1, 44)_ = 2.00, *p* = 0.164].

## Discussion

This study examined verbal production in people with schizophrenia and healthy controls in the context of a referential communication task based on the presentation of a series of movie characters. The movie characters were either very well-known characters that most women in their twenties would be expected to know (the Likely-Known condition), or characters that would be known by only a minority of women in their twenties according to prior surveys (the Likely-Unknown condition). A central finding from this study is that people with schizophrenia did not adjust the number of pieces of information that they provided when presenting the Likely-Known vs. the Likely-Unknown movie characters, contrary to the healthy controls who used more pieces of information to present the Likely-Unknown characters.

This result is consistent with a previous study that also relied on the referential communication paradigm and which observed a reduced adjustment to the known vs. unknown nature of the referents in people with schizophrenia (13). In that study, the authors focused on the production of indefinite vs. definite references and observed that SZ participants used a high proportion of indefinite references not only when introducing new items (as would be expected), but also when reintroducing the same items later during the task (in which case definite references would instead be expected). Participants with SZ in that study thus failed to mark the distinction between novel (unknown) vs. previously mentioned (known) referents through their use of indefinite vs. definite references, just like the SZ participants in the current study failed to mark the distinction between the Likely-Known vs. Likely-Unknown characters through the presentation of additional pieces of information.

In the study by Champagne-Lavau et al. (13), people with schizophrenia with greater ToM deficits showed even fewer adjustments in their use of reference markers. In our study, a significant correlation with ToM was observed only in our control group, such that heathy participants with better ToM performance showed a greater adjustment in the number of pieces of information that they used depending on the Likely-Known or Likely-Unknown nature of the characters [see also (22)]. This correlation was however absent (and even negative) in our SZ group, which likely reflects that we could not observe a correlation when there was no adjustment to start with. Even if ToM can help people make speech adjustments based on their interaction partner's likely knowledge, like in our healthy control group, the current results suggest that, in people with schizophrenia, good ToM abilities do not seem sufficient to promote similar adjustments.

Nonetheless, participants with SZ were not totally insensitive to the Likely-Known or Likely-Unknown nature of the characters as some significant effects of study conditions emerged across both groups when considering the *type of information* that was used to present the characters. More specifically, across both groups there were more Likely-Known trials for which participants used only movie-related information to present the characters, and more Likely-Unknown trials for which they used only descriptive information. This suggest that some form of adjustments did occur even in the SZ group.

The healthy controls however used description only for a very low proportion of trials (around 10%, see Figure 3), significantly less than in the SZ group. Instead, healthy controls more often used a combination of movie-related and descriptive information. The trials for which participants used a combination of movie-related and descriptive information involve, by definition, at least two pieces of information (one or more of each type). In contrast, the trials for which participants used only descriptive information could consist of the presentation of a single piece of information at least for some of the trials. The significant group by condition interaction that we observed for the number of pieces of information thus seems to reflect that the healthy participants used descriptive information *in addition to* movie-related information when faced with a Likely-Unknown character, whereas people with SZ used descriptive information *instead of* using movie-related information on those trials (i.e., they used descriptive information only).

It is useful to keep in mind that even for the Likely-Unknown characters, there was always a possibility that the matcher could know the characters and could potentially use the movie-related information to identify which character was being presented. Movie-related information was thus always potentially useful. But even in instances where the matcher cannot possibly know the material, previous studies have found that healthy participants still tend to name and describe the items on a significant proportion of trials (24). In the study by Heller et al. (24), pairs of participants learned together the names of a series of abstract shapes. One participant then learned additional names alone, before acting as the speaker in a referential communication task based on the presentation of the abstract shapes to the other participant (the matcher). The speakers in that study used a name and describe strategy (similar to our combination of movie-related and descriptive information) for a significant proportion of trials, especially for the condition in which the speakers had learned the names alone. Hence, naming or using movie-related information does not seem to reflect an improper impression that the matcher would know the material, and could rather serve other purposes such as informing the matcher about the speaker's knowledge (22), or teaching them the names for future use (24).

Given that our analyses focused specifically on the characters from movies that the participants had themselves seen, participants knew at least the titles of the movies, as attested by their responses to the movie knowledge questionnaire that they filled before performing the task. It is nonetheless possible that participants in the SZ group did not remember the name of the characters as well as the healthy controls did. People with SZ often present with memory deficits (36) and it is possible that these deficits led them to use descriptive information only for at least some of the trials.

For people with schizophrenia, using only descriptive information to present characters from movies that were known to them could give the false impression that the character was actually unknown to them, which could lead to their interaction partners potentially misjudging (underestimating) their level of knowledge and consequently impacting their real-life conversations. Though speculative, this is an interesting question that could certainly be targeted in future studies. Another point to explore in future studies is the link with alogia, a negative symptom often observed in SZ and characterized by a reduction in the quantity of speech. It is possible that people with SZ presenting with alogia may be particularly prone to using little information to present the characters, which could also lead to fewer adjustments in the number of pieces of information used to present the characters in the current task.

The limitations of the current study include the relatively small sample size, which may have limited our ability to detect significant interactions between groups and conditions in our analyses focusing on the types of information. For instance, the interaction was at *p* = 0.071 for the combination of movie information and descriptions and would have thus reached significance in the context of a unidirectional test (i.e., directional hypothesis that the SZ group adjusts less). It could be interesting to replicate the current results in a larger group of participants. Another limitation is that there was a very large proportion of male participants in our sample, which may limit the generalization of the results. Future studies should thus aim to include a greater proportion of females. Another limitation is the small number of trials in our experimental task, which could be strengthened in future studies. For example, a recent study by Achim et al. (37) used a strategy where 25 Likely-Known and 25 Likely-Unknown characters that the participants themselves knew were selected for each participant prior to the experimental task. The behavioral results from that functional MRI study in healthy participants closely replicated those of the healthy participants in the current study and in Achim et al. (22). The study by Achim et al. (37) also confirmed that it is feasible to adjust the task to maximize and balance the number of characters retained in the analyses for each condition of the task, with the only constraint that it takes a very large set of recently validated stimuli to reach 25 Likely-Unknown characters that the participants themselves know. The data from the present study were however acquired before this updated version of the task was designed.

## Conclusion

This study adds to our knowledge about referential choices in people with schizophrenia, showing that people with schizophrenia did not adjust the number of pieces of information that they provided depending on the likely knowledge of their interaction partner. Furthermore, this study raises an interesting new hypothesis about the lesser self-disclosure observed in the discourse of people with schizophrenia, which would certainly deserve further attention.

## Data availability statement

The raw data supporting the conclusions of this article will be made available by the authors, without undue reservation.

## Ethics statement

The studies involving human participants were reviewed and approved by Research Ethics Board of the CIUSSS-CN—Neuroscience and Mental Health Division. The patients/participants provided their written informed consent to participate in this study.

## Author contributions

AmA and MF designed and supervised the study. AmA and AnA completed the analyses. AmA drafted a first draft of the manuscript. AnA and MF helped improve the manuscript and approved the final version. All authors contributed to the interpretation of the results.

## Funding

This work was supported by a Social Sciences and Humanity Research Council of Canada (SSHRC) grant (#410-2011-1628), and the Fonds de Recherche du Québec—Santé supported our team through salary awards to AmA.

## Conflict of interest

The authors declare that the research was conducted in the absence of any commercial or financial relationships that could be construed as a potential conflict of interest.

## Publisher's note

All claims expressed in this article are solely those of the authors and do not necessarily represent those of their affiliated organizations, or those of the publisher, the editors and the reviewers. Any product that may be evaluated in this article, or claim that may be made by its manufacturer, is not guaranteed or endorsed by the publisher.
